# Characterization of Detailed Sedentary Postures Using a Tri-Monitor ActivPAL Configuration in Free-Living Conditions

**DOI:** 10.3390/s23020587

**Published:** 2023-01-04

**Authors:** Myles W. O’Brien, W. Seth Daley, Beverly D. Schwartz, Madeline E. Shivgulam, Yanlin Wu, Derek S. Kimmerly, Ryan J. Frayne

**Affiliations:** 1 School of Physiotherapy (Faculty of Health) & Division of Geriatric Medicine (Faculty of Medicine), Dalhousie University, Halifax, NS B3H 4R2, Canada; 2 Geriatric Medicine Research, Dalhousie University & Nova Scotia Health, Halifax, NS B3H 4R2, Canada; 3 Division of Kinesiology, School of Health and Human Performance, Faculty of Health, Dalhousie University, Halifax, NS B3H 4R2, Canada

**Keywords:** lying time, bent-knee sitting, inclinometry, sitting postures, body positions

## Abstract

Objective monitors such as the activPAL characterize time when the thigh is horizontal as sedentary time. However, there are physiological differences between lying, bent-legged sitting, and straight-legged sitting. We introduce a three-monitor configuration to assess detailed sedentary postures and demonstrate its use in characterizing such positions in free-living conditions. We explored time spent in each sedentary posture between prolonged (>1 h) versus non-prolonged (<1 h) sedentary bouts. In total, 35 healthy adults (16♀, 24 ± 3 years; 24 h/day for 6.8 ± 1.0 days) wore an activPAL accelerometer on their thigh, torso, and shin. Hip and knee joint flexion angle estimates were determined during sedentary bouts using the dot-product method between the torso–thigh and thigh–shin, respectively. Compared to lying (69 ± 60 min/day) or straight-legged sitting (113 ± 100 min/day), most time was spent in bent-legged sitting (439 ± 101 min/day, *p* < 0.001). Most of the bent-legged sitting time was accumulated in non-prolonged bouts (328 ± 83 vs. 112 ± 63 min/day, *p* < 0.001). In contrast, similar time was spent in straight-legged sitting and lying between prolonged/non-prolonged bouts (both, *p* > 0.26). We document that a considerable amount of waking time is accumulated in lying or straight-legged sitting. This methodological approach equips researchers with a means of characterizing detailed sedentary postures in uncontrolled conditions and may help answer novel research questions on sedentariness.

## 1. Introduction

Sedentary is defined as any awake, low-energy-expenditure (<1.5 metabolic equivalents of task) behavior in a sitting, reclining, or lying posture [1]. National [2] and international [3] activity guidelines have evolved from being solely grounded in physical activity intensity to focusing on a whole-day approach that emphasizes limiting total sedentary time, as well as prolonged sedentary bouts (e.g., >1 h uninterrupted). Evidence regarding the negative health consequences of excessive sedentary time, particularly for prolonged sedentary bouts, is growing [4]. The development of movement guidelines are largely based on population-wide observational studies that implement self-reported or device-based measures of sedentary time [5]. Device-determined sedentary time is commonly measured by thigh-worn accelerometers that distinguish upright time from sedentary postures [6]. Using accelerometers to measure habitual postures may be more cumbersome than self-report questionnaires but addresses the limitation of participant recall bias and potential inaccuracies attributed to poor question interpretation [7]. As technological advancements in hardware and software occur, it is expected that the measurement of sedentary postures will be more refined and will improve researcher’s ability to answer novel research questions regarding the negative health consequences of sedentariness.

Sedentary time is an all-encompassing term to describe time spent in sitting, reclining, or lying postures [1]. However, laboratory-based studies have identified divergent physiological responses to each of these postures. For example, lower-limb vascular function [8] and arterial blood pressure [9] are lower in a sitting versus lying posture. Furthermore, it has been postulated that part of the negative vascular consequences of prolonged sitting are attributed to knee joint angles, with prolonged bent-leg lying provoking worse vascular outcomes than straight-leg lying [10]. Given the differences in physiological responses between sedentary postures [8,9] and the documented impact of knee angle [10], partitioning specific sedentary postures in free-living conditions would be an important step forward in characterizing habitual body positions. The utility of dual-monitor configurations to broadly identify sitting versus lying has been established [11,12]. This entailed positioning accelerometers on the thigh and torso simultaneously and has been implemented in laboratory or short-duration free-living environments (<24 h) [11]. The dual-monitor configuration helped researchers identify whether a participant’s torso was vertical (i.e., sitting) or horizontal (i.e., lying), when their thigh was horizontal, permitting additional insight into their sedentary posture. Given that participant activity measurements are typically conducted over multiple days in uncontrolled conditions, understanding the feasibility of the measurement tools in these conditions and time windows is important. Currently, a habitual monitoring surveillance system that provides further detailed sedentary posture characteristics does not exist. A measurement of habitual sitting time would equip physiologists with the ability to extend their laboratory studies to free-living environments and assist the development of sitting-specific public health guidelines.

We propose that monitors positioned concurrently on the thigh, torso, and shin of an individual can provide additional detailed information on body postures, such as time spent in various hip and knee joint angles. The purpose of this study was to explore specific hip and knee joint flexion angles over the course of a free-living habitual monitoring period. We compared time spent in habitual lying, bent-legged sitting, and straight-legged sitting postures and explored whether these varied between prolonged versus non-prolonged sedentary bouts. Additionally, we provided our analysis program that calculates these angles using the commonly implemented activPAL monitor [13] to assist researchers interested in characterizing detailed sedentary postures to answer their own novel research questions.

## 2. Materials and Methods

Thirty-five healthy, young adults (<40 years, 16 non-pregnant females) consented to participate in the study. All participants were non-hypertensive (seated resting systolic blood pressure ≤ 139 and diastolic blood pressure ≤89 mmHg via automated vital signs monitor [Carescape V100; General Electric Healthcare, Mississauga, ON, Canada]). Some thigh-only activPAL data (3 males, 8 females) have been previously presented evaluating the impact of habitual activity on vascular function [14]. Height and weight were measured using a calibrated stadiometer/physician’s scale (Health-O-Meter, McCook, IL, USA) to the nearest 0.5 cm and 0.1 kg, respectively. These measures were then used to calculate body mass index. Prior to testing, verbal and written informed consent were acquired. All protocols and procedures conformed to the Declaration of Helsinki and were approved by the Dalhousie University Health Sciences Research Ethics Board (#2020-5214 (approved August 2017, 2020) and #2021-5555 (approved 25 May 2021)). All participants provided written informed consent.

**Figure 1:**
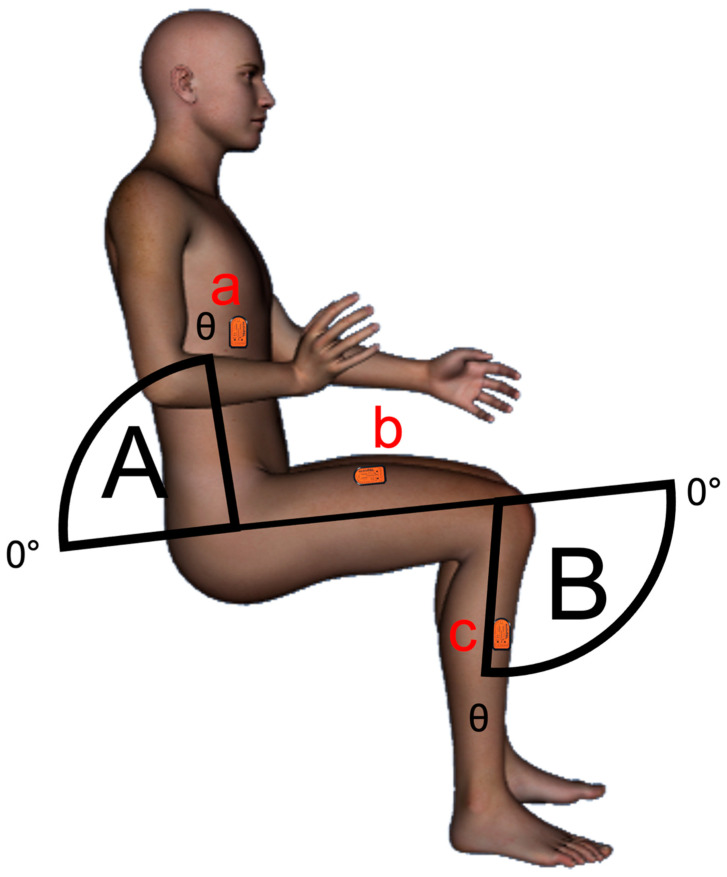
A depiction of activPAL monitor placements on the torso (a), thigh (b), and shin (c). The hip and knee flexion angles were calculated from the torso–thigh (A) and thigh–shin (B).

Participants were equipped with three activPAL inclinometers (PAL Technologies Ltd., Glasgow, UK) positioned on their torso, thigh, and shin that were waterproofed via a nitrile finger cot and secured using Tegaderm^TM^ medical dressing (3M, London, ON, Canada) [13]. Based on recommended guidelines [13], the thigh monitor was positioned on the right anterior thigh, one third of the way between the hip and the knee. The other two monitors were positioned on the anteromedial side of the right tibia and the right side of the torso, parallel to the other two monitors, just below the ribcage (see Figure 1). Participants wore the activPAL monitor 24 h per day for a minimum of 5 days including one weekend day (mean ± SD: 6.8 ± 1.0 days) [15]. Participants self-reported their waking hours via a diary of wake-up and sleep times to distinguish bouts of sleep from sedentary time. Bouts during self-reported sleep times were excluded from the analyses.

The activPALs were synchronized to start recording simultaneously at midnight of the first day of monitoring. It has been demonstrated that sensor drift over time is not a concern with the activPAL monitors when recording free-living activity [16]. Acceleration data were sampled at 20 Hz, downloaded, and processed (PALanalysis V7) to provide “.csv” files, which were used in a custom program to calculate hip and knee flexion angles (as described below). The acceleration data were low-pass filtered at a 0.18 Hz cut-off (i.e., 1st order zero-lag digital Butterworth filter) [17]. ActivPAL versions 3 s and 4 s were utilized, but all tri-monitor configurations included the same versions within participants. ActivPAL 4 s has a higher recording capacity (14 vs. 7 days) and resolution (10 bits/sample with ±4 g range vs. 8 bits/sample with ±2 g range), but resolution is a minor concern for stationary postures, and our program is built for either version.

The sedentary joint-angle position algorithm was developed using MATLAB (R2022a, The MathWorks Inc., Natick, MA, USA). The analysis program is available from GitHub [18]. The raw XYZ acceleration.csv files for each activPAL monitor (thigh, torso, and shin) were extracted from the default PALanalysis V7 software. The “events” file, which denotes the start and duration of each bout of activity to the nearest 0.1 s from the thigh monitor was also extracted from PALanalysis. From the events file, the MATLAB program identified bouts that the thigh monitor characterized as sedentary, then extracted the corresponding accelerations from the thigh, torso, and shin raw XYZ acceleration files. It has been reported that activity ≤20° from horizontal is characterized as sedentary by the activPAL [12].

Relative hip flexion angles and knee flexion angles between activPALs were determined during the sedentary bouts using the dot-product method [Equation (1)] between the torso–thigh and thigh–shin, respectively. Hip and knee joint angles, relative to the standard anatomical position, and the duration of each event were separated into 15° bins (to characterize joint postures throughout a full range of motion) and exported to a .csv file. The time of day for each sedentary bout was also exported to facilitate the isolation of sedentary time and removal of sleep. Apart from the selection of the specific files, no researcher input was required to analyze joint angle data.

(1) $$Joint\;Angle=\cos^{-1}\left(\frac{acceleration\;vector\left(a\right)}{\sqrt{X_{a}{}^{2}+Y_{a}{}^{2}+Z_{a}{}^{2}}}\cdot \frac{acceleration\;vector\left(b\right)}{\sqrt{X_{b}{}^{2}+Y_{b}{}^{2}+Z_{b}{}^{2}}}\right)$$

All data are presented as means ± standard deviation. Time spent in each degree bin is presented descriptively for knee and hip flexion angles. Bin sizes in 15° were selected based on our preliminary, unpublished results observing a mean absolute error of ~5° compared to motion capture for knee joint angles >15°, whereas this error increases to ~10° at angles <15°. Hip angles <30° were considered lying, and angles >30° were considered sitting for the purpose of this study. Within sitting, knee angles >45° and <45° were conservatively classified as bent-knee and straight-legged sitting, respectively. A higher knee-angle degree indicates greater knee flexion. Definitions of what angle constitutes sitting versus lying or bent-knee versus straight-legged are unclear, but the angles selected serve as likely reasonable heuristic thresholds in the absence of recommendations. Given the documented physiological differences [19] and separate international guidelines [2,3] for prolonged versus non-prolonged sedentary time, we examined time spent in sedentary postures separately for prolonged (i.e., >1 h) versus non-prolonged sedentary time (i.e., <1 h). In the absence of a strict definition of what is “prolonged”, 1 h was selected due to the established negative impact of sedentary bouts >1 h on leg blood flow [10], endothelial function [19], and arterial blood pressure [20].

Normality was assessed via a Shapiro–Wilk test, and each sedentary position was determined to be non-normal (all, *p* < 0.002). Accordingly, Wilcoxon-signed rank tests compared time spent in bent-legged sitting, straight-legged sitting, and awake lying time. The *p*-value was adjusted for multiple comparisons (*p* = 0.05 ÷ 3 comparisons = 0.017). Similarly, time spent in prolonged bouts was compared against that spent in non-prolonged bouts within each sedentary posture via Wilcoxon-signed rank tests. All statistical analyses were completed in SPSS (Version 28, IBM Corp., Armonk, NY, USA).

## 3. Results

There were 239 total days of observations included in this study. Participants were 24 ± 3 years (range: 19–31) and had a body mass index of 24.4 ± 3.4 kg/m^2^ (17.7–30.3).

Average time spent in specific knee and hip angles for prolonged bouts (>1 h), non-prolonged bouts (<1 h), and combined sedentary events is presented in Table 1. The length of bouts <1 h per participant was 8.8 ± 1.8 min (range: 5.2–12.6 min). In general, during sedentary bouts, more time was spent in the knee and hip flexion angles >75°. In total, participants accumulated an average of 621 ± 130 min/day of sedentary time, with more time accumulated in non-prolonged sedentary bouts (416 ± 93 min/day) versus prolonged sedentary bouts (206 ± 115 min/day).

**Figure 2:**
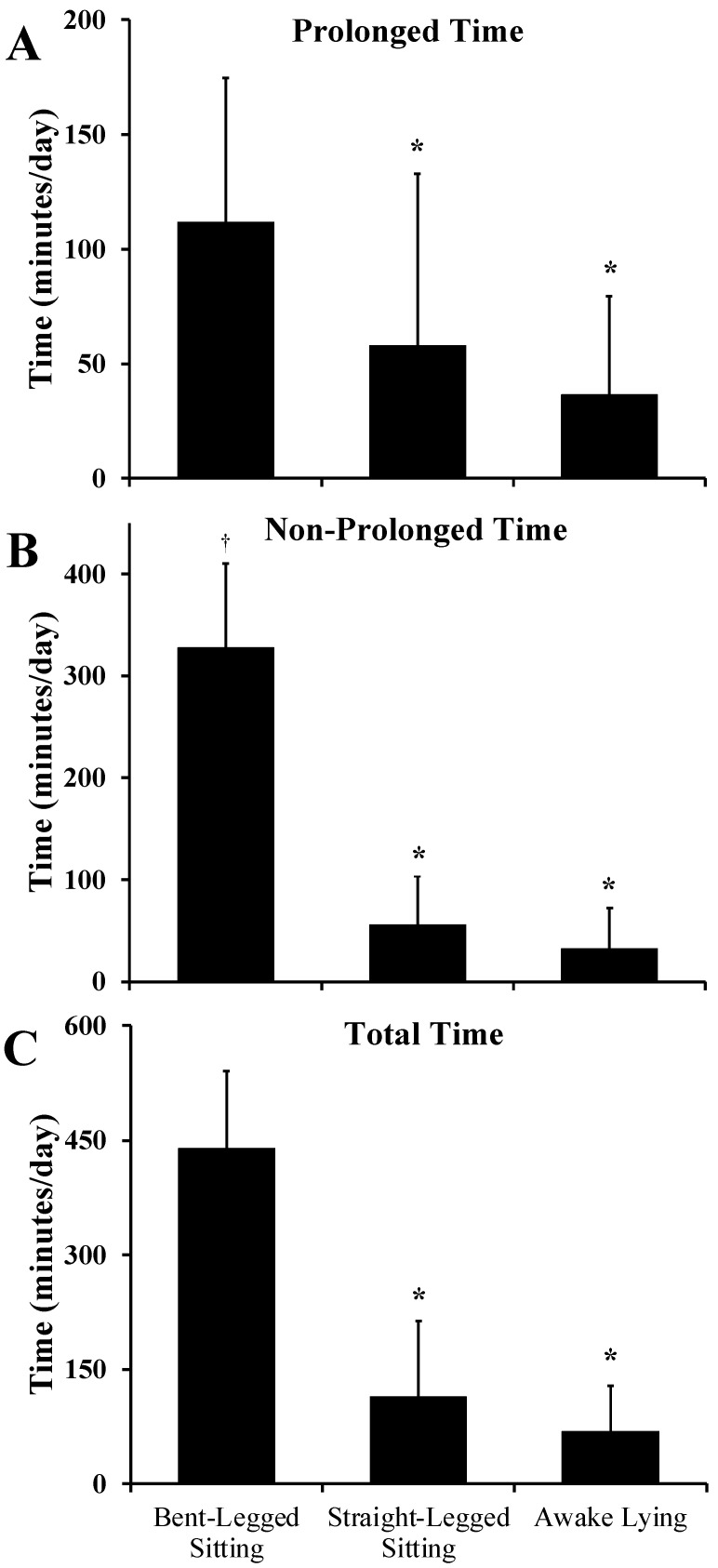
Time spent in bent-legged sitting, straight-legged sitting, and lying across prolonged sedentary bouts (>1 h; panel (**A**)), non-prolonged sedentary bouts (<1 h; panel (**B**)), and total time (panel (**C**)). Time across each position and between prolonged and non-prolonged bouts with each position were compared via a Wilcoxon-signed rank test. * *p* < 0.0167 (adjusted for multiple comparisons) to bent-legged sitting. † *p* **<** 0.0167 to prolonged bent-legged time.

As presented in Figure 2, most sedentary time was characterized as bent-legged sitting compared to straight-legged sitting and lying, independent of whether the bout was prolonged or non-prolonged (all, *p* < 0.001). Time in straight-legged sitting and lying were not different regardless of bout length (all, *p* > 0.09). Comparing prolonged versus non-prolonged bouts, more bent-legged sitting time was spent in non-prolonged bouts (*p* < 0.001). However, prolonged versus non-prolonged durations were not different for straight-legged sitting or lying (both, *p* > 0.26), as presented in Figure 2.

**Figure 3:**
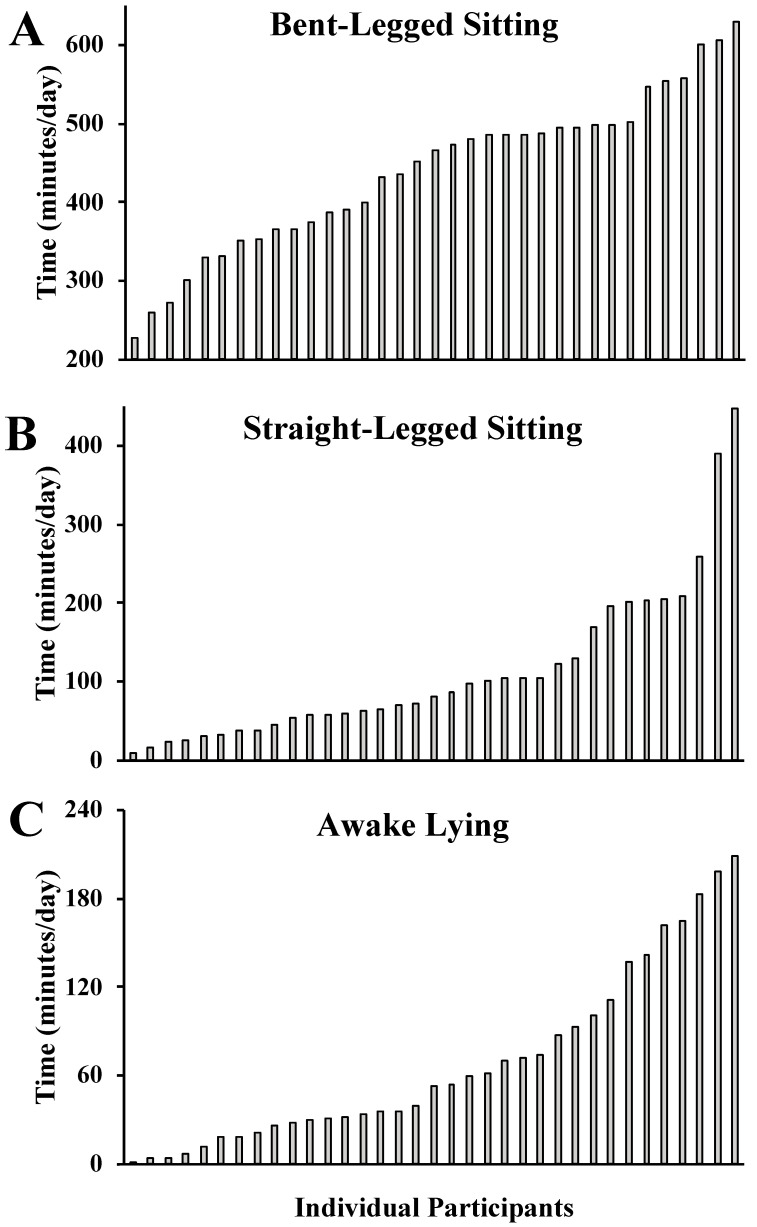
Waterfall plots presenting the individual average time within bent-legged sitting (panel (**A**)), straight-legged sitting (panel (**B**)), and lying (panel (**C**)). Participants were ordered from least-to-most amount of time separately within each position.

There was substantial inter-individual variability for how sedentary time was accumulated between postures, as presented in Figure 3. Average sedentary time between participants ranged from 227–629 min/day for bent-legged sitting, 9–457 min/day for straight-legged sitting, and 1–209 min/day for lying.

## 4. Discussion

The purpose of our study was to explore specific hip and knee joint flexion angles over the course of a free-living habitual monitoring period in healthy adults and provide an easy-to-use introductory program for researchers interested in advancing the field of sedentary behavior. We documented that most sedentary time was accumulated in bent-legged sitting, but a considerable amount (~30% of time) was accumulated in either straight-legged sitting or lying (Figure 2). However, there was a lot of variability in time spent in lying, bent-knee sitting, and straight-legged sitting between participants (Figure 3). The presented tri-monitor framework and analytical program advance our ability to measure specific habitual sedentary postures.

Emerging research has demonstrated the utility of dual-monitor configurations to distinguish lying from sitting, which is otherwise not possible or poorly estimated from single monitors [11,12,21,22]. Specifically, compared to direct observation, activPAL monitors positioned on the thigh and torso exhibited high sensitivity and specificity in characterizing sedentary bouts as sitting versus lying [12]. Our study adds to this literature by expanding from broadly defined sitting versus lying postures and introducing a method that better characterizes sedentary positions by estimating specific hip and knee flexion angles. The innovative approach used in the present study implements multiple sensors to quantify lying vs. knee-bent sitting vs. straight-legged sitting. Without the presented surveillance system and approach, it is not possible to accurately characterize these different positions in free-living conditions. The presentation of the utility of this novel surveillance strategy and the accompanying code in quantifying these behaviors has the capacity to greatly improve the measurement of specific sedentary positions by researchers worldwide beyond single monitor and self-report measures. Furthermore, we extend this broad characterization to differentiating prolonged versus non-prolonged bouts of sedentary time, with evidence accumulating that prolonged bouts are associated with adverse health outcomes independent of total sedentary time [23,24]. Together, presenting specific joint angles and the consideration of bout lengths provides a useful addition to the literature that may be used by health, exercise science, and applied researchers to design novel research studies and questions regarding sedentariness. Appreciating the disagreements [25,26] of how sedentary time is included in existing movement guidelines, the methodological information that can be attained by a free-living tri-monitor configuration may be used to develop more refined sedentary behavior guidelines that consider the time in nuanced postures.

Bent-legged sitting time was the most frequent sedentary posture, but there was a considerable amount of time accumulated in lying and straight-legged positions, which may be the result of our conservative definition of straight-legged sitting (<45°). However, our data provide evidence that not all sedentary time is accumulated in a similar posture and that ~30% of sedentary time was accumulated in non-bent-legged sitting (18% straight-legged sitting, 12% lying; Figure 2). Accordingly, the common description of thigh-worn accelerometry determined sedentary time as “sitting” may be confounded by awake lying time, which was quite substantial among some individuals (i.e., range: 1–209 min/day; Figure 3). Dual-monitor or tri-monitor configurations are clearly needed to truly measure sitting time, with single monitors’ abilities to distinguish sitting from lying exhibiting modest validity [11]. Consideration of these heterogeneous sedentary patterns may be informative for sedentary researchers, especially if the negative impact of sedentary positions is specific to one of its encompassing behaviors. For example, it is reasonable to hypothesize that the health impacts of sitting may differ from lying given the posture-specific physiological effects (e.g., blood pressure [9]) of body position.

The overarching methodological approach presented may help with the design of targeted interventions or the characterization of body postures performed by clinical populations (e.g., hospital inpatients), whereby patients who are bed-ridden are encouraged to sit up more than stay lying during their hospital stay. In addition, a greater knee bend during sitting may be associated with augmented reductions in leg blood flow versus sitting with straighter legs [10]. However, our understanding of the vascular responses to prolonged sitting is largely limited to laboratory settings [27,28]. Our study and the accompanying software equip physiology researchers with the ability to expand such studies from the laboratory to a free-living environment.

Modern science is progressing towards better open access that encourages transparent research practices [29]. Although existing dual-monitor approaches have been utilized [11,12,21,22], there is minimal guidance for researchers and/or software programmers to utilize such analysis programs. Our study advances this aspect of open science in the measurement of sedentary time by including our analysis program for researchers to derive detailed sedentary postures. As described in detail within existing reviews on the topic [30,31,32], the validity of monitors to detect sedentary positions is important and well documented, but researchers may be interested in sitting time specifically. Our provided analysis program helps address this gap and provides researchers with the capacity to isolate sitting time from lying time, which may be useful for developing activity monitoring devices and/or validating sitting-time questionnaires. Although we provide our program in the MATLAB computing language, some researchers may not have access to this software. However, our program may be converted to other computer languages by researchers interested in this area of research. Overall, this transparent sharing of our analysis program should be used to modify and further advance the measurement of sedentary postures. This will better facilitate the refinement of future analytical and surveillance strategies as advancements in technology progress.

The absolute times in each body position is specific to our sample of younger adults and may vary in more sedentary, older populations [33], who may engage in more daily lying time than younger adults. Future studies understanding detailed sedentary postures in office workers, older, and/or clinical populations (e.g., hospital in-patients) warrants further investigation. The degree of participant burden of wearing two additional monitors on their torso and shin are unclear and worthy of future study. The method used to calculate joint angles is sensitive to rotations along the long axis and, therefore, future work should consider analytical strategies for correcting or eliminating sedentary positions in which gravity is acting predominantly on the medio-lateral axis (e.g., cross-legged sitting, side-lying, etc.). Furthermore, postural adjustments while in a sedentary state (defined by a horizontal thigh), will create movement accelerations that will increase the error of measurement; therefore, future studies should identify the frequency, duration, and effect of sedentary postural adjustments on estimates of hip and knee flexion using this analysis method. The definition of sedentary time incorporates a metabolic aspect (i.e., <1.5 metabolic equivalents of task) [1]. As with any accelerometer, we are unable to discern this component, and it is possible that monitors may have incorrectly characterized exercise in a sitting or lying posture (e.g., bench press) as sedentary. Certainly, the program and ideas presented are a starting point for further validation studies and analytical programs. Lastly, we used the frequently implemented activPAL monitors [13,31] in this study and rely on their acceleration outputs, but the principles implemented into our program could be applied to other accelerometers.

## 5. Conclusions

Thigh-worn activity monitors characterize all horizontal positions as the same sedentary posture, but the definition of sedentary time comprises sitting, reclining, and lying positions. We used a combination of torso, thigh, and shin monitors to estimate knee and hip flexion angles in free-living sedentary conditions and equip researchers with the program necessary to conduct such posture analysis. These more detailed sedentary postures may be used to improve our understanding of sedentary physiology and answer novel research questions regarding the effects of sedentary postures in uncontrolled environments.

## Figures and Tables

**Table 1:** Time spent in specific knee flexion angles and hip flexion angles during prolonged, non-prolonged and total sedentary bouts.

Position	Prolonged Sedentary Bouts (min/day)	Non-Prolonged Sedentary Bouts (min/day)	Total Sedentary Bouts (min/day)
Knee Angle			
<15°	16.3 ± 25.8	14.0 ± 15.6	30.3 ± 34.97
15–30°	32.4 ± 34.0	28.6 ± 18.7	61.0 ± 43.8
30–45°	33.6 ± 29.5	35.7 ± 23.2	69.2 ± 44.0
45–60°	7.5 ± 6.8	17.6 ± 13.4	25.1 ± 15.5
60–75°	26.3 ± 23.8	53.6 ± 24.0	79.8 ± 37.0
>75°	71.7 ± 40.5	230.3 ± 60.0	302.0 ± 77.1
Hip Angle			
<15°	11.8 ± 13.9	10.6 ± 21.2	22.4 ± 27.7
15–30°	33.3 ± 31.4	26.6 ± 21.9	59.9 ± 43.6
30–45°	37.1 ± 33.9	42.1 ± 26.4	79.2 ± 46.2
45–60°	11.3 ± 8.1	21.3 ± 8.7	32.6 ± 12.6
60–75°	31.4 ± 18.1	78.9 ± 37.8	110.4 ± 42.1
>75°	53.0 ± 32.8	195.2 ± 98.3	248.2 ± 113.9

Data presented as mean ± SD. Prolonged sedentary bouts were defined as >1 h. Time was averaged into min/day across all days. Days without a prolonged sedentary bout >1 h result in 0 min for that day; this explains the average time in prolonged sedentary bouts being <1 h.

## Data Availability

All data files can be provided by the corresponding author, M.W.O., upon reasonable request.
